# *In situ* Detection of Microbial Life in the Deep Biosphere in Igneous Ocean Crust

**DOI:** 10.3389/fmicb.2015.01260

**Published:** 2015-11-12

**Authors:** Everett C. Salas, Rohit Bhartia, Louise Anderson, William F. Hug, Ray D. Reid, Gerardo Iturrino, Katrina J. Edwards

**Affiliations:** ^1^Jet Propulsion Laboratory, Planetary Chemistry and Astrobiology, California Insitute of TechnologyPasadena, CA, USA; ^2^Photon Systems, Inc.Covina, CA, USA; ^3^Department of Geology, University of LeicesterLeicester, UK; ^4^Lamont-Doherty Earth Observatory, Marine Geology and GeophysicsPalisades, NY, USA; ^5^Department of Biological Sciences and Earth Sciences, University of Southern CaliforniaLos Angeles, CA, USA

**Keywords:** deep subsurface biosphere, microbial life, deep UV fluorescence, *in situ* life detection, North Pond, borehole

## Abstract

The deep biosphere is a major frontier to science. Recent studies have shown the presence and activity of cells in deep marine sediments and in the continental deep biosphere. Volcanic lavas in the deep ocean subsurface, through which substantial fluid flow occurs, present another potentially massive deep biosphere. We present results from the deployment of a novel *in situ* logging tool designed to detect microbial life harbored in a deep, native, borehole environment within igneous oceanic crust, using deep ultraviolet native fluorescence spectroscopy. Results demonstrate the predominance of microbial-like signatures within the borehole environment, with densities in the range of 10^5^ cells/mL. Based on transport and flux models, we estimate that such a concentration of microbial cells could not be supported by transport through the crust, suggesting *in situ* growth of these communities.

## Introduction

The deep marine biosphere is reportedly a massive scale suite of microbial provinces (Schrenk et al., 2010). It is known that microbial life is metabolically active (D'Hondt, 2002), to varying degrees, and extends at least 1.6 km into subsurface sediments (Roussel et al., 2008; D'Hondt et al., 2009). By comparison with what we know for the sedimentary deep biosphere habitats, however, very little is known about the size, function, and activity of microbial life in igneous ocean crust—or even if it truly exists. Igneous ocean crust harbors the largest aquifer on Earth, most of which is hydrologically active (Stein and Stein, 1994; Fisher and Becker, 2000), providing the possibility that microbial life may extend well underground in this habitat as well.

In microbial ecology, scientists are challenged to make even the most basic microbial cell enumerations—this is particularly true in the igneous ocean crust as cells likely exist in biofilms associated with volcanic surfaces in the porous aquifer. The diminutive size and transparency of microbes necessitates the use of stains and fluorescence (Daley and Hobbie, 1975; Giovannoni et al., 1988) for detection on opaque surfaces, such as igneous crust. However, while these methods have enabled detection, visualization and some characterization, the organic and chemical variability of natural samples can lead to confounding results where tags and stains non-specifically bind to background structures (Nadeau et al., 2008). Further, the substrates can produce their own fluorescence from the near UV (>300 nm) and visible excitation wavelengths used to excite the tags, leading to false positives (Bhartia et al., 2010).

We have developed and applied a novel tool for the *in situ*, rapid detection of microorganisms in the igneous marine deep biosphere by capitalizing on intrinsic fluorescence of bound and free aromatic amino acids; tryptophan, phenylalanine, and tyrosine. Excitation with a < 250 nm source enables a means to use these compounds to detect potential signatures of microbial life in these environments. Deep UV excitation results in fluorescence spectral features that can be used to distinguish biological samples from other naturally occurring or anthropomorphically generated fluorophores. This capability to differentiate microbial cells from organic matter is a result of a cell having a high concentration of proteins containing strongly fluorescing bound aromatic amino acids, free amino acids, redox-active membrane proteins and other aromatic compounds (e.g., flavins, NADH). While some bacterial cells can extend the range of fluorescence to beyond 400 nm, most of the unique fluorescence signature associated to all known bacterial cells or spores ranges between 270 and 400 nm. The spectral feature is similar, but not identical to the fluorescence feature of tryptophan, tyrosine, or phenylalanine and can be differentiated from organic matter in the environment (Bhartia et al., 2008, 2010). The Deep Exploration Biosphere Investigative tool (DEBI-t), is based on the use of deep ultraviolet (< 250 nm) native fluorescence for rapid, non-contact detection and localization of microbial communities against opaque mineralogical backgrounds (Bhartia et al., 2010). Although the DEBI-t instrument does not have the spatial resolution to determine whether detected spectral signatures are definitively cellular features, the bulk signature analysis shows a consistent ability to differentiate between organics and lab grown microbes and microbes from the terrestrial environment.

**Figure 1:**
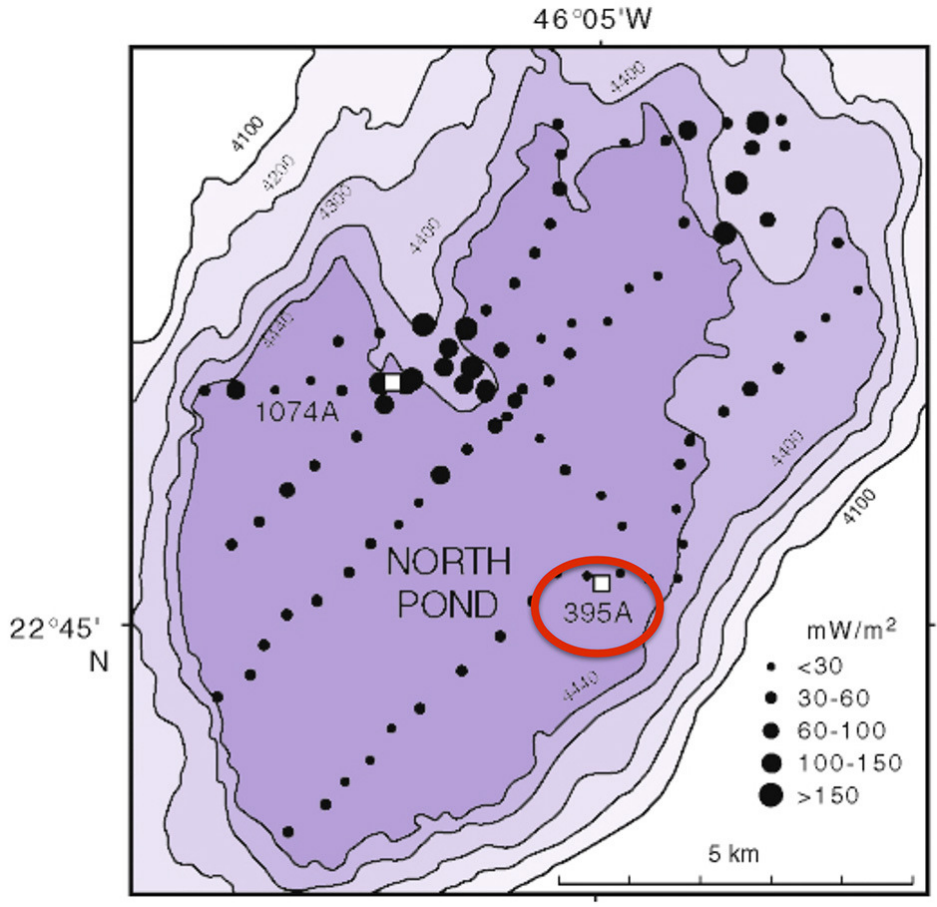
**Bathymetric map of North Pond showing the location of Hole 395A**. The black circles represent a heat flow survey conducted during DSDP 78B (Langseth et al., 1992). Hole 395A was drilled during DSDP 45, and is located about 1 Km from the southeastern edge of North Pond (red circle).

DEBI-t was deployed as a wireline tool via logging operations in Hole 395A, a Deep Sea Drilling Program legacy borehole that was drilled in 1974-75. Hole 395A, located at the North Pond site in the western flank of the Mid-Atlantic Ridge (Figure 1), was sealed at the seafloor using a circulation obviation retrofit kit (CORK; Becker and Davis, 2005) in 1998 (Becker et al., 2001), and then reopened in September 2011 during IODP Expedition 336 with the goal of deploying DEBI-t and installing a modernized CORK.

DEBI-t relies on a deep UV (< 250 nm) laser induced native fluorescence spectroscopy, which enables the detection of microbial cells and organics without tagging or staining.

DEBI-t utilizes a 224.3 nm HeAg hollow cathode laser used to induce fluorescence of organics and microbes. The instrument uses a “soft pulse” of 600 nJ/100 μs pulse to avoid damage or alteration of the materials and operates at a frequency of 4 Hz. The exits the instrument via a deep UV transmissive sapphire window, with a 20 mm clear aperture, seated at the front of the housing. A turning mirror within the sonde directs light to and from the sidewall (Figure S1). Fluorescence emissions are detected using a 150 mm focal length lens coupled to with linear dichroic filter stack that splits the collected light into 7 spectral bands, each with a discrete photomultiplier tube (PMT) coupled to a filter and focusing lens. The optical design has a depth of field from the wall of the instrument to 16^′′^ from the instrument. The bandpass filters associated with each PMT was used to detect fluorescence emissions from the 280 to 455 nm with 20 nm bandwidths (full-width-half-max). The center wavelengths are set at 280, 300, 320, 340, 360, 380, and 455 nm. The instrument is also equipped with a pinhole camera that records video of the downhole logging operation to provide spatial context to the fluorescence information. Additional tool specifications are given in Table S1.

## Results

DEBI-t was deployed as part of what was designated the microbiology combination tool string, with sondes that recorded 3-axis downhole acceleration, 3-axis magnetic field, temperature and total and spectral (Th, U, K) gamma-ray measurements (Figure S2). These measurements were used to correlate fluorescence data to physical properties collected for Hole 395A during previous expeditions. The instrument utilized a “fire and forget” methodology, in which the embedded control software directed the instrument to fire the laser, collect data and transmit information uphole when power was supplied. Power and real-time telemetry were supplied via a 24 V power supply in the multi-function telemetry module (MFTM). During logging operations, DEBI-t transmitted real-time clock, laser power and detector status to provide the operators on the ship with information regarding downhole *in situ* conditions.

The microbiology combo string was deployed immediately after removal of the old CORK, to minimize introduction of bottom water contaminants. During logging operations at Hole 395A, the instrument acquired native fluorescence data during two complete passes of the borehole, where logs were obtained in both the downhole and uphole directions. Comparison of DEBI-t with mid-Atlantic ocean water and bacterial standards showed a unique spectral signature for the borehole data that was distinct from ocean water (Figure S3).

**Figure 2:**
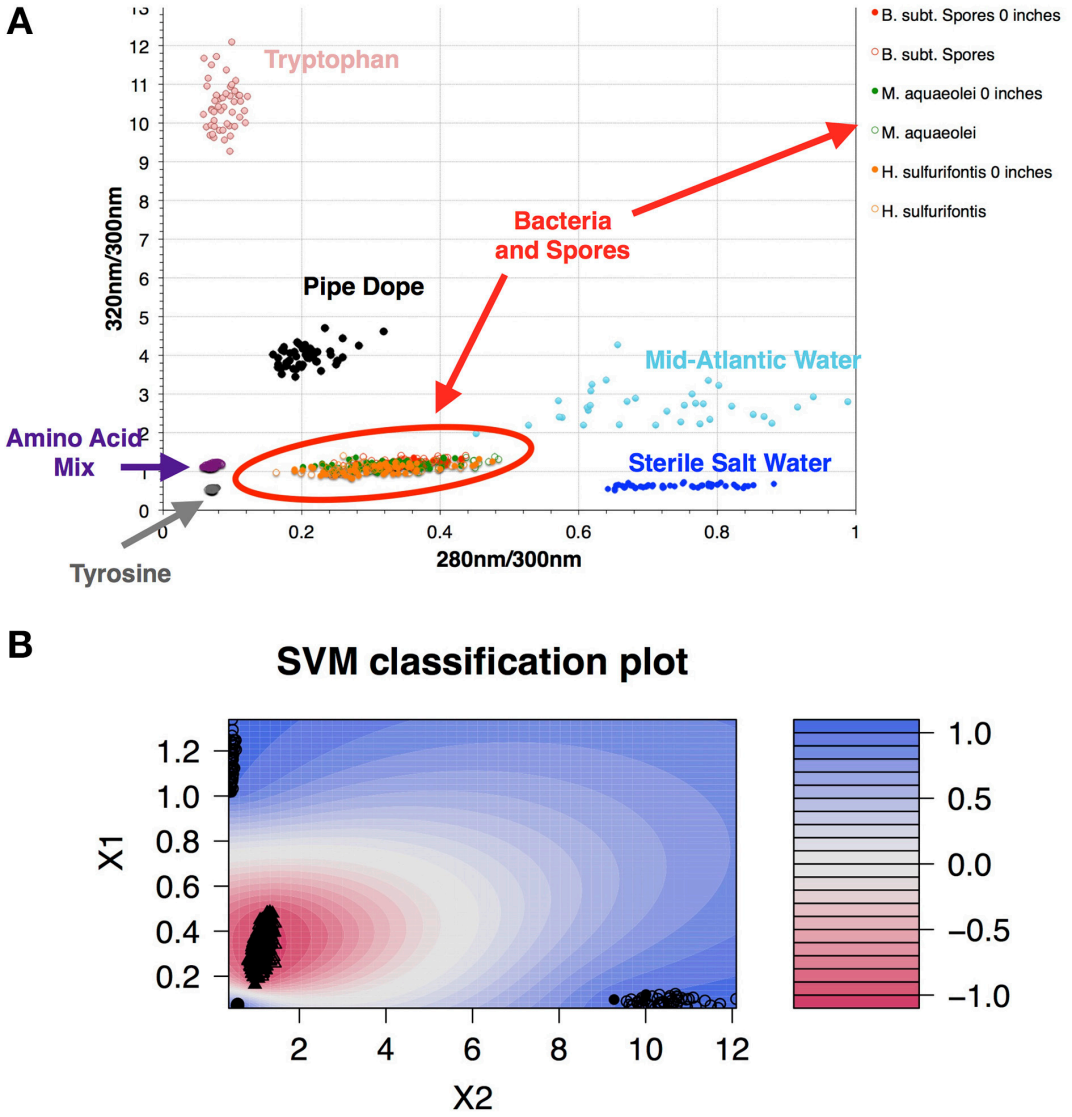
**A library of organics and microbes was used to classify data collected at Hole 395A**. The database was classified in a binary form: “microbe” and “organic.” **(A)** Scatter plot of the standards used for classifying the data collected with DEBI-t. Because the instrument had a long depth of focus, the data was collected at two distances: just in front of the turning mirror (0 inches); and 10 inches from the turning mirror. Fluorescence spectra from all bacterial standards collected at both distances clustered in the same location (red oval); **(B)** SVM contour plot with the fitted decision values using the library. A semi-supervised Laplacian kernel was used to train the SVM. The scale (right) shows the decision boundary values for the two sets, with −1 denoting the “most microbe-like,” and +1 denoting the “most organic-like,” or “least microbe-like.”

**Figure 3:**
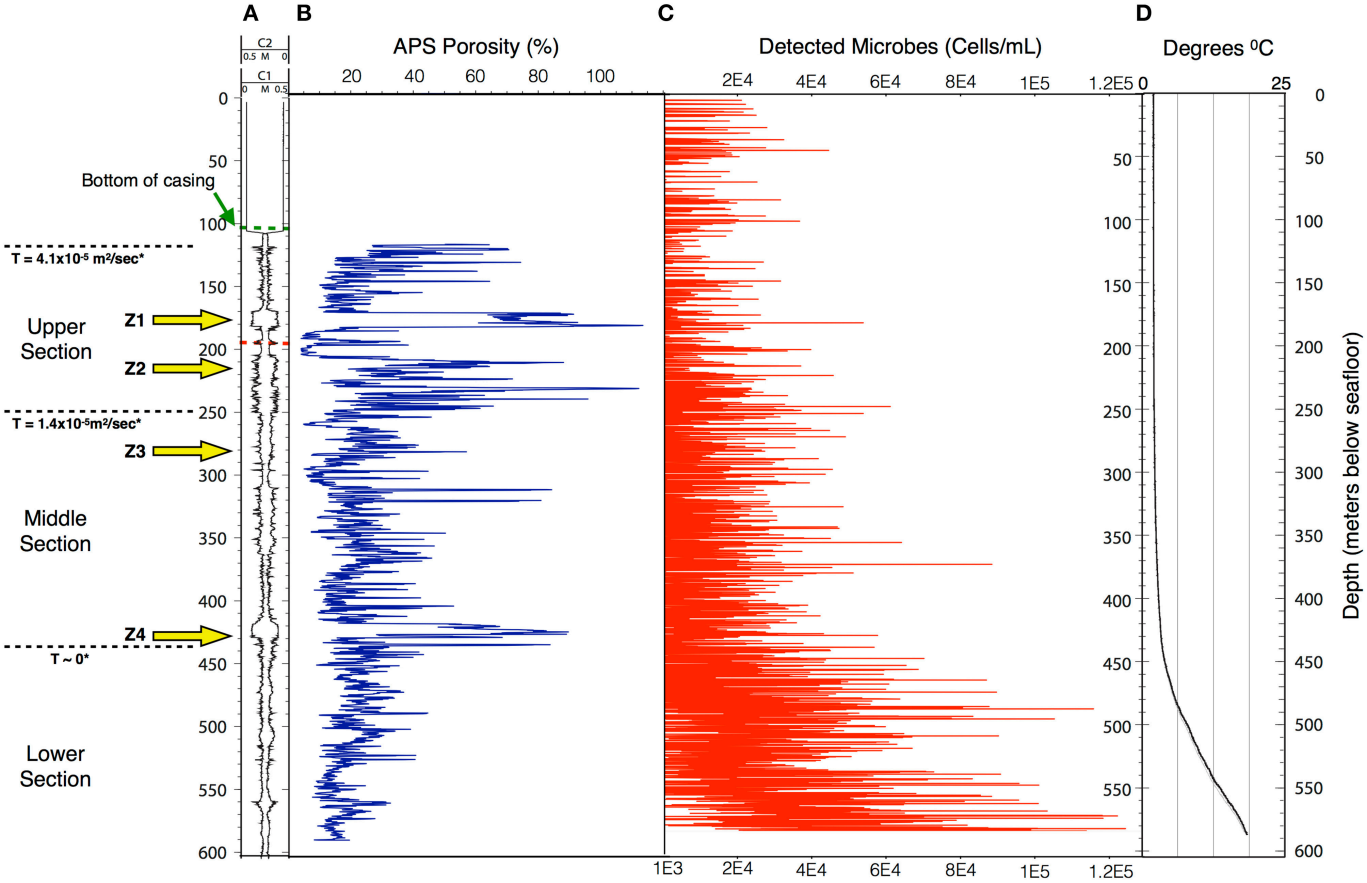
**The structure of the microbe-like signature within Hole 395A appears to be indicative of settling**. The overall density of microbes increases with depth in Hole 395A. **(A)** Caliper data taken for Hole 395A during ODP Leg 174B; C1 = FMS pass 1, C2 = FMS pass 2; green hash line = location of bottom of casing, red hash line = location of drill pipe for second logging attempt during IODP Expedition 336; location of inflow zones (yellow arrows) is based on spontaneous potential log from Hole 395A collected during ODP Leg 174B; T = hydraulic transmissivity from Morin et al. (1992); based on transmissivity values, the hole was portioned into three sections. **(B)** APS porosity data collected during ODP Leg 174B. **(C)** Scatter plot showing the density range of detected spore signals during the first downlog; the density range is dependent on the excitation source's depth of penetration through the water column; red circles = upper estimate, black circles = lower estimate.; for modeling purposes. **(D)** Temperature profile of Hole 395A. Data is from ODP Leg 174B and IODP Expedition 336. The temperature profile suggests a hydrologically isolated lower portion, which would be consistent with the observed structure of the microbial signatures.

Data collected in 395A was analyzed using a Support Vector Machine (SVM). SVMs are supervised learning algorithms, which find an optimum hyperplane that maximizes the separation, or margin, between two classes. The function used to describe this hyperplane can be specified by a training set, known as support vectors. For this investigation, the two classes used were “microbe” and “non-microbe.” A library of “microbe” data was constructed from spectral information collected using bacteria, bacterial spores and archaea, while tryptophan, phenylalanine and tyrosine mixtures were used to construct the “non-microbe” dataset. The library data used to train the SVM was acquired using the DEBI-t with the same acquisition parameters used for the logging data. Because the chemical information in the standards produced a library that was non-linear (Figure 2) a Laplacian kernel was used to optimize the hyperplane. The DEBI-t logging data from 395A was then input into the SVM to be classified as “microbe” or “non-microbe.” A conservative value of 95% confidence was used categorize data points as microbial. Results of logging data for Hole 395A demonstrated a significant presence data points that were consistent with microbial signals. Data for the first downhole pass indicated an increase in the detected biomass, with the biggest change occurring below 450 m below sea floor (Figure 3). Data from the subsequent uphole and downhole passes showed an increased homogenization of this signal, suggesting disturbance of the hole resulting from the tool string's movement through the borehole. The collected video data (Movie M1) showed an abundance of orange particulate material within the borehole, which may be interpreted as aggregated iron hydroxide particles and microbial flocs.

## Discussion

Using published data for the fluorescence cross-section of microbes with deep UV excitation and the instrument optical parameters (Faris et al., 1997; Bhartia et al., 2010), we can estimate that the bioload within Hole 395A is approximately 10^4^–10^5^ cells per mL (Figure 3), which is greater than the reported cell density of the overlying bottom water (Meyer et al., 2012). The calculated density is dependent on the effective view volume of the instrument. Although DEBI-t has a nominal view volume of 1–2 ml assuming the full depth of field of the instrument. However, particulate matter, evident in the video data collected in line with the spectral data (Movie M1), indicate that the effective view volume was significantly reduced and a majority of the signal was collected from around the focused spot, which we estimate to be between ca. 10 ~ 200 uL. This suggests that the bulk of the signal originated within the water column, not the wall, and were either planktonic microbes or attached to the particulates.

To ascertain the possibility that the detected signals were the result of cells transported through the crust, a simple box model was used to estimate the discharge of cells into the borehole. The transport and filtration of microbes through subsurface environments has been extensively studied, but the factors contributing to this process are still poorly constrained (Tufenkji, 2007). Several aspects can influence adhesion of a microbe onto a surface: substrate surface charge, microbial-surface zeta potential, nutrient availability, fluid flow, among others (Harvey et al., 2002; Tufenkji, 2007; Boks et al., 2008). However, while transport and accumulation behavior is still under intensive investigation, classical colloid filtration theory (CFT) is currently used by researchers to model and evaluate microbial subsurface transport (Ginn et al., 2002; Tufenkji, 2007). Under CFT, adhesion of bacteria onto a surface can be reduced to a few parameters that make up the following equations:

(1) $$\begin{array}{ccc} \ln\;(C_{x}) & = & \ln\;(C_{0})-K \end{array}$$

(2) $$\begin{array}{ccc} K & = & \frac{K_{d}L}{V} \end{array}$$

(3) $$\begin{array}{ccc} K_{d} & = & \frac{3}{2}\frac{\left(1-\theta \right)}{d}\eta \alpha U \end{array}$$

where *C*_*x*_ = concentration of particles at some distance from the recharge zone, *C*_0_ = the concentration of particles at the recharge zone, L = distance from the recharge zone, V = laminar flow velocity of the fluid, θ = porosity, d = grain diameter, η = contact efficiency, i.e., the rate at which a particle strikes a substrate divided by the rate at which the particle moves toward the substrate, α = attachment/filtration efficiency, i.e., the ratio between the number of collisions that succeed in producing attachment of a particle to a substrate divided by the total number of collisions, K_*d*_ = filtration coefficient, K = the reduction in particle number, and U = particle velocity/substrate porosity. In these models, adhesion is considered to be irreversible (Yao et al., 1971; Chang et al., 1983; Tong et al., 2005; Tufenkji, 2007). Assuming a particle diameter of 1 μm, η can be estimated to be approximately 10^−2^–10^−3^ (Yao et al., 1971; Harvey and Garabedian, 1991), while fieldwork has produced values of α on the order of 7 × 10^−3^ for microbes (Harvey and Garabedian, 1991; Schijven et al., 2000). Filtration efficiencies for endospores are, on average, about 1/3 those of vegetative cells (Chang et al., 1983; Schijven et al., 2000). It is unclear where the recharge zones for North Pond are located; however, subsurface flow is thought to flow from southeast to northwest (Becker et al., 2001, 2012). For simplicity, we assume that recharge occurs in the exposed basalt approximately 1100 m to the southeast of North Pond. Finally, cell concentrations in Mid-Atlantic Ocean Bottom Water are estimated to be on the order of 10^4^ cells/mL (Meyer et al., 2012).

**Figure 4:**
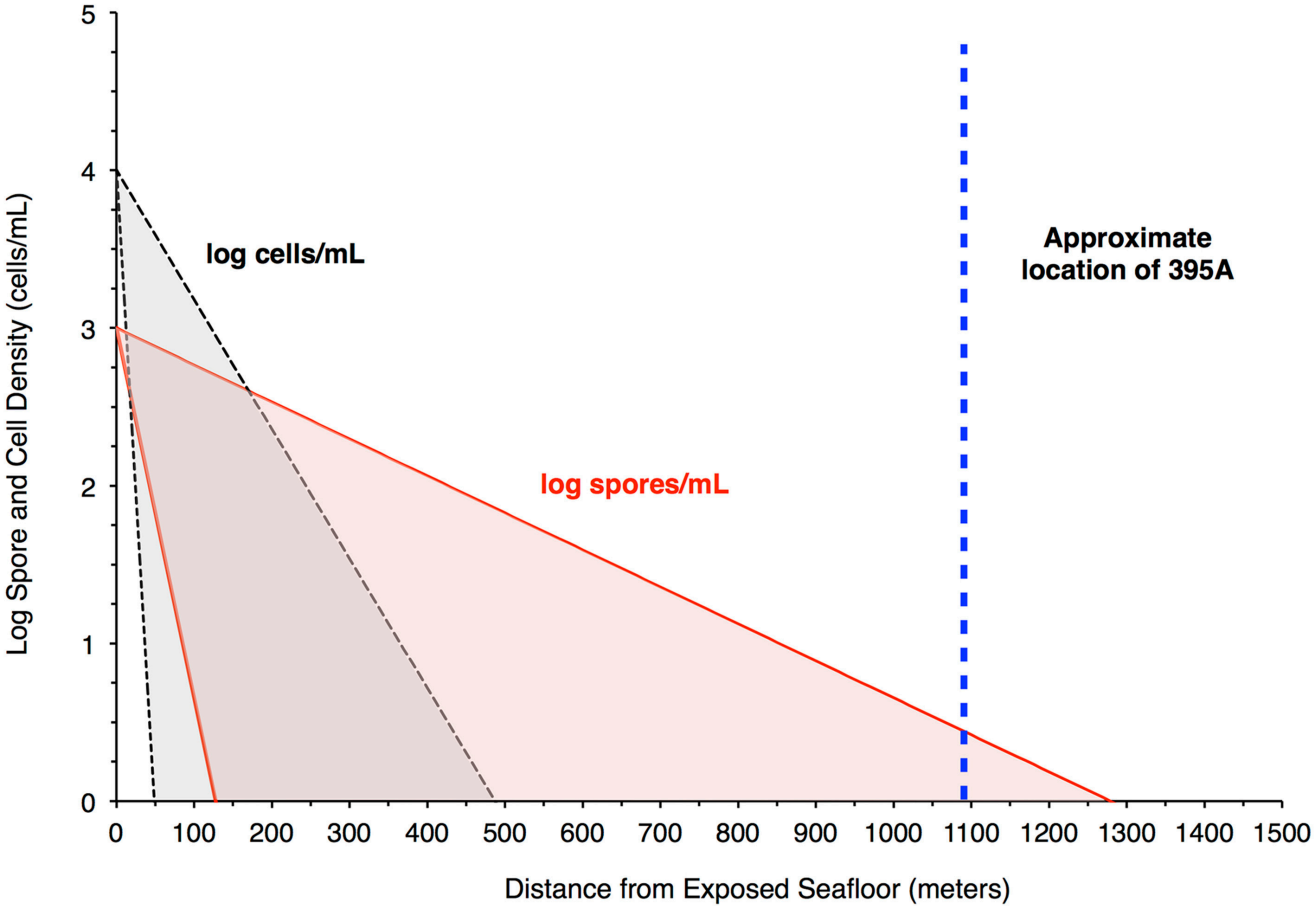
**Filtration of bacteria in the crust would produce higher spore densities in pore water 1 Km from a putative recharge zone**. The plot shows results from a model that attempts to assess the likelihood of seeing a greater concentration of spores relative to bacteria in pore water at the location of Hole 395A. The red shaded area bordered by the red solid lines indicates the concentration of spores in pore fluids, while the gray shaded area bordered by the black hashed lines indicates the concentration of cells in pore fluids. The blue, hashed line is the location of Hole 395A relative to the exposed ocean crust southwest of North Pond, which serves as a putative recharge zone for purposes of modeling. The model suggests that spore densities will drop off less rapidly than cell densities in the pore fluids.

The resulting filtration model suggests that cells would be filtered out of the porewater long before arrive at the location of 395A (Figure 4). Although the lack of constraints leads to a wide scope of concentrations as a function of distance, the general trend remains the same. Given the known hydrology of Hole 395A (Langseth et al., 1984; Morin et al., 1992; Becker et al., 2001), it was assumed that the bulk of fluids discharged into the hole came from 4 inflow zones (Figure 3). A simple box model (Supplementary Material) was constructed to estimate the expected concentration of cells within the borehole after 13 years of isolation from the overlying bottom water (Table 1). Fluid velocities through these inflow regions were estimated to be approximately 100 m per year (Wheat, personal communication). Results from these models suggest that transport could not lead to the observed cell densities (Figure 3).

**Table 1:** **Assumptions used for calculating microbial transport model**.

**Estimated cell density in Mid-Atlantic Ocean Bottom Water (cells/mL)**	~10^4^^*^
**Estimated spore density in Mid-Atlantic Ocean Bottom Water (spores/mL)**	~10^3^^*^
ESTIMATED CELL FLUX FROM EACH INFLOW ZONE (cells^*^m^−2^^*^yr^−1^)	
**Inflow Zone 1**	**2.4 × 10^8^**
**Inflow Zone 2**	**1.5 × 10^8^**
**Inflow Zone 3**	**1 × 10^8^**
**Inflow Zone 4**	**6 × 10^7^**
ESTIMATED SPORE FLUX FROM EACH INFLOW ZONE (spores^*^m^−2^^*^yr^−1^)	
**Inflow Zone 1**	**2.4 × 10^9^**
**Inflow Zone 2**	**2.4 × 10^9^**
**Inflow Zone 3**	**2 × 10^9^**
**Inflow Zone 4**	**1.5 × 10^9^**
Estimated vertical velocity for spores and cells (m^*^yr^−1^)	31.5^**^

* *Cell densities are estimated from work carried out during Maria S. Merian Expedition 20/5 (Meyer et al., 2012). Discharge numbers for cells and spores were calculated using estimates from the transport model and laminar flow calculations based on data collected during ODP Leg 174B*.

** *Vertical transport velocity calculated from published work measuring settling rates of spore and spore-like particles (Kempf et al., 2005)*.

**Figure 5:**
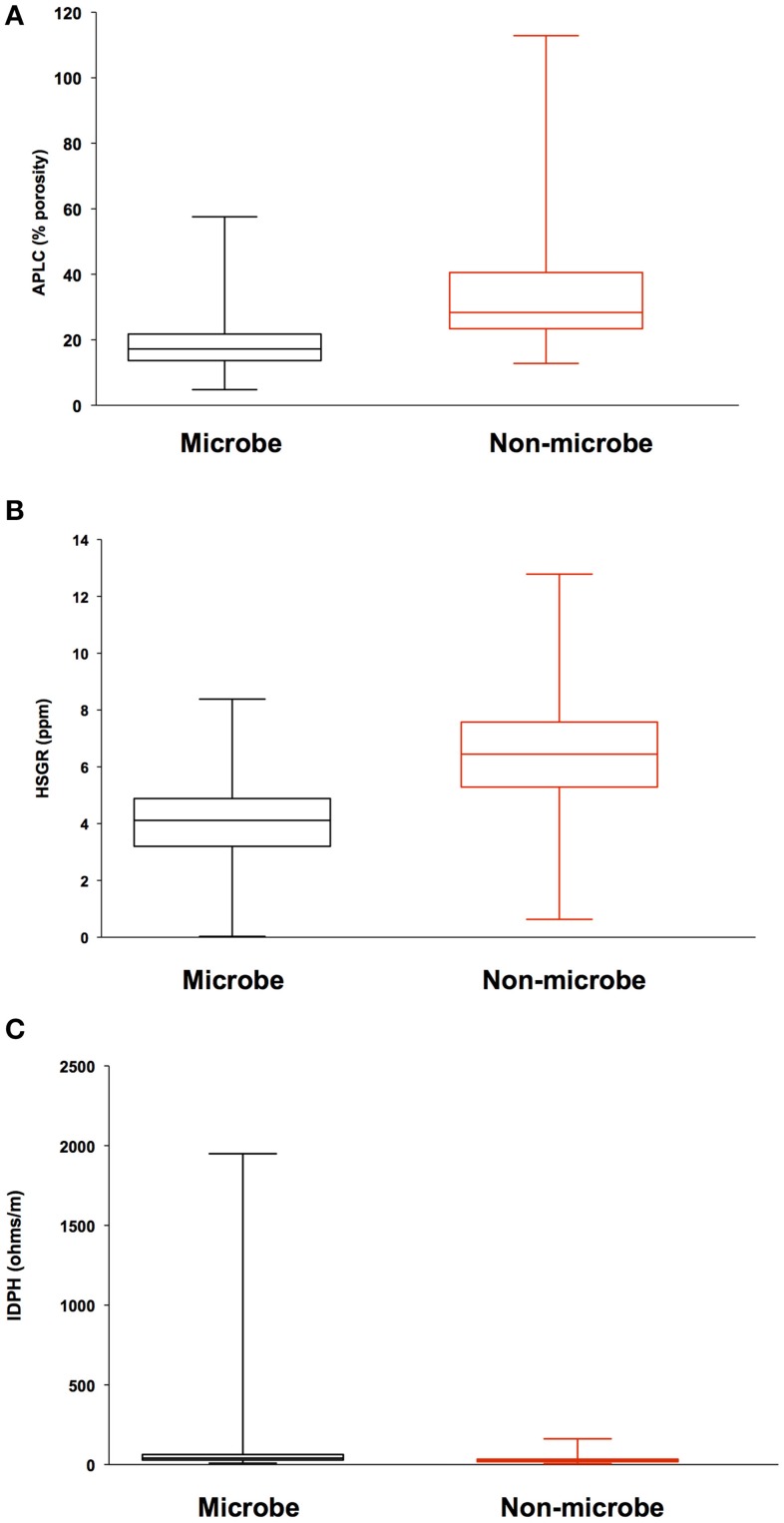
**Iterative Non-hierarchical Cluster Analysis (INCA) indicates no relationship between the structure of the detected microbial signatures and the lithological properties within 395A**. Briefly, INCA is a variation on K-means clustering, in which objects are moved into and out of clusters, the number of which has been defined by the user (Harvey et al., in prep). The objective is to minimize the variability within clusters, while maximizing the variability between clusters. Resistivity, porosity, and gamma ray profiles for 395A were compared to the distribution of microbial signatures to determine if there was a relationship between the shape of the microbial data and the lithology of 395A. Results from INCA indicate no relationship between the observed structure of the microbial signals, and the lithology of 395A. Data are presented as box and whisker plots for simplicity, and indicate overlap with all physical properties data between the two categories (microbe and non-microbe) used in the analysis. **(A)** Accelerator porosity sonde; provides porosity information for the borehole wall; **(B)** hostile natural gamma ray sonde; used to provide information on lithology, e.g., rock vs. clay; **(C)** dual induction resistivity tool; provides spontaneous potential data that can be used to elucidate density, porosity, and lithologic boundaries.

Analysis of geophysical data also failed to correlate a relationship between areas of high inflow along the borehole sidewall and locations where biomass was detected. Porosity, density, and total gamma ray values where compared to microbial signals from DEBI-t using an iterative, non-hierarchical cluster analysis (Figure 5). Areas of higher porosity, lower density, and lower gamma radiation would be indicative of regions with potentially higher lateral fluid flow (Crosby and Anderson, 1971; Helm-Clark et al., 2004). However, the variability in these parameters did not correlate with detected microbial fluorescence signals.

An alternative possibility is that the detected microbes were produced, *in situ*, by active communities residing along the borehole wall. In this case, the transport of microorganisms through the igneous crust could lead to the establishment of communities located within the outflow zones. Although the communities located on the outer walls would have been scraped off during drilling and subsequent reaming, the communities within the crust surrounding the borehole should have remained intact. Further, introduction of the CORK would have provided another substrate for microbes to attach to. The removed CORK was well oxidized, and given the microbial genomic data collected from other basalt zones (Emerson et al., 2007), there is the possibility that iron oxidizers were able to use the CORK as a potential energy source. If the detected signals within Hole 395A represent cells that detached and subsequently reattached to particles within the borehole water column, then the population attached to the walls or CORK may be on the order of 10^4^–10^5^ cells/cm^3^ in the upper portion of the hole, with the lower portion being comprised of material which settled to the bottom. Vertical transport of particle-attached microbes would be consistent with what is known about the physical nature of 395A, as the lowest 150 m of the hole is thought to be relatively impermeable (Langseth et al., 1992).

The presence of microbial cells in igneous oceanic crust has been previously inferred from petrographic observations of alteration textures in basaltic glass (Fisk et al., 1998), measurements of the presence of chemicals that are consistent with biology (carbon, nitrogen, phosphorous), observed the presence of morphological features interpreted as bacterial cell shapes (Banerjee and Muehlenbachs, 2003), and via molecular analysis of cored materials (Bach and Edwards, 2003). However, neither chemical nor trace fossil evidence can unequivocally prove the existence of microbial life in igneous ocean crust, nor demonstrate the timing of any inferred microbiological activities. Previous theoretical work on bioenergetics of alteration reactions has suggested that microbial communities should be supported as a result of water-rock reactions that occur in young igneous oceanic crust (Bach and Edwards, 2003). Despite these evidences, prior data establishing the *in situ* presence of microbial biomass in the oceanic crustal environment has been scant. Three previous studies have recovered, isolated, and analyzed DNA data from rocks recovered in the Atlantic and Pacific Oceans (Bach and Edwards, 2003; Mason et al., 2010; Santelli et al., 2010) and one has analyzed similar data from novel *in situ* rock colonization experiments conducted in young igneous oceanic crust (Orcutt et al., 2010). These studies have not established the ubiquity or abundance of life in the crust.

The deep marine biosphere has been hypothesized to potentially harbor up to 2/3 of the prokaryotic biomass on Earth, or 1/3 of the total biomass carbon (Whitman et al., 1998). Recent studies have called into question such estimates (D'Hondt et al., 2007; Kallmeyer et al., 2012), pointing out the fact that most data collected for the deep marine biosphere have been rendered largely from continental slopes and margins, which are organic-rich provinces on a global basis compared to the volumetrically more abundant open ocean provinces. Furthermore, it has been suggested that activity levels for the deep marine biosphere are exceptionally slow, with potential turnover rates of 1000 years (Jørgensen and D'Hondt, 2006). It is important to recognize, however, that all data collected to date that are considered as part of this ongoing census of deep life derives from marine sediments. No studies have considered cellular abundances or activities in the hard rock provinces below deeply buried sediments. It has been hypothesized, however, that any biosphere in the igneous ocean crust would have to be characterized by exceptionally low abundances and low activity owing to the fact that energy and nutrient resources in these provinces should be low (Jørgensen and D'Hondt, 2006). However, one recent study examining colonization of native rocks and minerals in the crust as well as the data presented herein suggest another interpretation is possible, and that the igneous oceanic crust may represent a vibrant and dynamic deep biosphere (Orcutt et al., 2010). Further studies that employ technologies that enable enumeration and activity measurements in other crustal provinces are required to address this enigma.

## Materials and methods

### Bacterial cultures

Bacteria were inoculated from frozen stocks into Luria-Bertani (LB) broth and grown overnight at 25°C, shaking at approximately 125 rpm. Archaeal cultures were provided by Victoria Orphan at Caltech. Spore samples were prepared as described in Kwan et al. (2011) and Kempf et al. (2005). All samples were washed 3 times in sterile 1% saline solution to remove all traces of growth media and other organics. Cells were then placed into 100 mm cuvettes containing a sterilized salt solution (36 per ml) at concentrations of approximately 10^5^ cells/mL for data collection using DEBI-t.

### Amino acid standards

HPLC-grade amino acids (Sigma-Aldrich) were used to create the amino acid standards. 1 mM stocks of tryptophan and tyrosine were prepared using 18 MOhm water. These stocks were then diluted to 100 uM concentrations in 100 mm cuvettes containing sterilized salt water.

### Bench setup for library standards

Because the penetration depth of the excitation source was not certain, a 100 mm cuvette was used to collect spectral information for standards (Figure S4). Data was collected at two positions; right behind the turning mirror, and 10 inches from the turning mirror. This was to ascertain any potential variation in the structure of the fluorescence spectra as a function of being in front of or behind the focal point. Result from this work indicated that there was no significant variation in the signals (Figure 2).

## Significance statement

This study describes the predominance of microbes within a marine borehole and postulates as to the potential source of this biomass. To the best of our knowledge, it represents the first set of microbial data collected *in situ*, and in real-time.

## Dedication

This work is dedicated to the memory of Katrina Edwards and Gerardo (Gerry) Iturrino. The successful deployment of this instrument would not have been possible without their efforts. We are glad to have known them as colleagues and friends. Although we mourn their passing, we celebrate their lives and contributions, whose impacts will be felt for years to come.

### Conflict of interest statement

The authors declare that the research was conducted in the absence of any commercial or financial relationships that could be construed as a potential conflict of interest.

## References

[B1] Bach W. Edwards K. J. (2003). Iron and sulfide oxidation within the basaltic ocean crust: implications for chemolithoautotrophic microbial biomass production. Geochim. Cosmochim. Acta 67, 3871–3887. 10.1016/S0016-7037(03)00304-1

[B2] Banerjee N. R. Muehlenbachs K. (2003). Tuff life: bioalteration in volcaniclastic rocks from the Ontong Java Plateau. Geochem. Geophys. Geosyst. 4, 1037 10.1029/2002GC000470

[B3] Becker K. Bartetzko A. Davis E. E. (2001). Leg 174B synopsis: revisiting Hole 395A for logging and long-term monitoring of off-axis hydrothermal processes in young oceanic crust. Proc. Ocean Drill. Progr. Sci. Res. 174B, 1–13. 10.2973/odp.proc.sr.174b.130.2001

[B4] Becker K. Davis E. E. (2005). A review of CORK designs and operations during the Ocean Drilling Program. Proc. Integr. Ocean Drill. Progr. 301, 1–28. 10.2204/iodp.proc.301.104.2005

[B5] Becker K. Villinger H. W. Davis E. E. (2012). Initial Pressure Data from the IODP Expedition 336 CORKs at North Pond. 2012 American Geophysical Union (San Francisco, CA), 1–2.

[B6] Bhartia R. Hug W. F. Salas E. C. Reid R. D. Sijapati K. K. Tsapin A.. (2008). Classification of organic and biological materials with deep ultraviolet excitation. Appl. Spectrosc. 62, 1070–1077. 10.1366/000370208786049123 18926014

[B7] Bhartia R. Salas E. C. Hug W. F. Reid R. D. Lane A. L. Edwards K. J.. (2010). Label-free bacterial imaging with deep-UV-laser-induced native fluorescence. Appl. Environ. Microbiol. 76, 7231–7237. 10.1128/AEM.00943-10 20817797 PMC2976275

[B8] Boks N. P. Norde W. Van der Mei H. C. Busscher H. J. (2008). Forces involved in bacterial adhesion to hydrophilic and hydrophobic surfaces. Microbiology 154, 3122–3133. 10.1099/mic.0.2008/018622-0 18832318

[B9] Chang P. W. Findley J. E. Yen T. F. (1983). Selection of bacteria with favorable transport properties through porous rock for the application of microbial-enhanced oil recovery. Appl. Environ. Microbiol. 46, 1066–1072. 16346414 10.1128/aem.46.5.1066-1072.1983 PMC239520

[B10] Crosby J. W. Anderson J. V. (1971). Some applications of geophysical well logging to basalt hydrogeology. Ground Water 9, 12–20. 10.1111/j.1745-6584.1971.tb03562.x

[B11] Daley R. J. Hobbie J. E. (1975). Direct counts of aquatic bacteria by a modified epifluorescence technique. Limnol. Oceanogr. 20, 875–882. 10.4319/lo.1975.20.5.0875

[B12] D'Hondt S. (2002). Metabolic activity of subsurface life in deep-sea sediments. Science 295, 2067–2070. 10.1126/science.1064878 11896277

[B13] D'Hondt S. Abrams L. Ferdelman T. G. Fischer J. Hasiuk F. Kallmeyer J. (2007). Life in subseafloor sediments of the South Pacific Gyre. AGU Fall Meeting Abstracts 1:08.

[B14] D'Hondt S. Spivack A. J. Pockalny R. Ferdelman T. G. Fischer J. P. Kallmeyer J.. (2009). Subseafloor sedimentary life in the South Pacific Gyre. Proc. Natl. Acad. Sci. U.S.A. 106, 11651–11656. 10.1073/pnas.0811793106 19561304 PMC2702254

[B15] Emerson D. Rentz J. A. Lilburn T. G. Davis R. E. Aldrich H. Chan C.. (2007). A novel lineage of proteobacteria involved in formation of marine fe-oxidizing microbial mat communities. PLoS ONE 2:e667. 10.1371/journal.pone.0000667 17668050 PMC1930151

[B16] Faris G. W. Copeland R. A. Mortelmans K. Bronk B. V. (1997). Spectrally resolved absolute fluorescence cross sections for bacillus spores. Appl. Opt. 36, 958–967. 10.1364/AO.36.000958 18250761

[B17] Fisher A. T. Becker K. (2000). Channelized fluid flow in oceanic crust reconciles heat-flow and permeability data. Nature 403, 71–74. 10.1038/47463 10638753

[B18] Fisk M. R. Giovannoni S. J. Thorseth I. H. (1998). Alteration of oceanic volcanic glass: textural evidence of microbial activity. Science 281, 978–980. 10.1126/science.281.5379.978 9703510

[B19] Ginn T. R. Wood B. D. Nelson K. E. Scheibe T. D. Murphy E. M. Clement T. P. (2002). Processes in microbial transport in the natural subsurface. Adv. Water Resour. 25, 1017–1042. 10.1016/S0309-1708(02)00046-5

[B20] Giovannoni S. J. DeLong E. F. Olsen G. J. Pace N. R. (1988). Phylogenetic group-specific oligodeoxynucleotide probes for identification of single microbial cells. J. Bacteriol. 170, 720–726. 2448289 10.1128/jb.170.2.720-726.1988 PMC210714

[B21] Harvey R. W. Garabedian P. S. (1991). Use of colloid filtration theory in modeling movement of bacteria through a contaminated aquifer. Environ. Sci. Technol. 25, 178–185 10.1021/es00013a021

[B22] Harvey R. W. Harms H. Landkamer L. (2002). Transport of Microorganisms in the Terrestrial Subsurface: In situ and Laboratory Methods. Manual of Environmental Microbiology, 2nd Edn. Washington, DC: ASM Press.

[B23] Helm-Clark C. M. Rodgers D. W. Smith R. P. (2004). Borehole geophysical techniques to define stratigraphy, alteration and aquifers in basalt. J. Appl. Geophys. 55, 3–38. 10.1016/j.jappgeo.2003.06.003

[B24] Jørgensen B. B. D'Hondt S. (2006). Ecology. A starving majority deep beneath the seafloor. Science 314, 932–934. 10.1126/science.1133796 17095684

[B25] Kallmeyer J. Pockalny R. Adhikari R. R. Smith D. C. D'Hondt S. (2012). Global distribution of microbial abundance and biomass in subseafloor sediment. Proc. Natl. Acad. Sci. U.S.A. 104, 16213–16216. 10.1073/pnas.1203849109 22927371 PMC3479597

[B26] Kempf M. J. Chen F. Kern R. Venkateswaran K. (2005). Recurrent isolation of hydrogen peroxide-resistant spores of Bacillus pumilus from a spacecraft assembly facility. Astrobiology 5, 391–405. 10.1089/ast.2005.5.391 15941382

[B27] Kwan K. Cooper M. La Duc M. T. Vaishampayan P. Stam C. Benardini J. N.. (2011). Evaluation of procedures for the collection, processing, and analysis of biomolecules from low-biomass surfaces. Appl. Environ. Microbiol. 77, 2943–2953. 10.1128/AEM.02978-10 21398492 PMC3126404

[B28] Langseth M. G. Hyndman R. D. Becker K. Hickman S. H. (1984). The hydrogeological regime of isolated sediment ponds in mid-oceanic ridges. Init. Rep. Deep Sea Drill. Proj. 78, 825–837.

[B29] Langseth M. G. Becker K. Herzen Von R. P. Schultheiss P. (1992). Heat and fluid flux through sediment on the western flank of the Mid-Atlantic Ridge: a hydrogeological study of North Pond. Geophys. Res. Lett. 19, 517–520. 10.1029/92GL00079

[B30] Mason O. U. Nakagawa T. Rosner M. Van Nostrand J. D. Zhou J. Maruyama A.. (2010). First Investigation of the microbiology of the deepest layer of ocean crust. PLoS ONE 5:e15399. 10.1371/journal.pone.0015399 21079766 PMC2974637

[B31] Meyer J. L. Jaekel U. Girguis P. R. Glazer B. T. Huber J. A. (2012). Microbial life in Cold, Hydrologically Active Oceanic Crustal Fluids. 2012 American Geophysical Union (San Francisco, CA), 1–2.

[B32] Morin R. H. Hess A. E. Becker K. (1992). *In situ* measurment of fluid flow in DSDP Holes395A and 534A: results from the dianaut program. Geophys. Res. Lett. 19, 509–512. 10.1029/91GL02947

[B33] Nadeau J. L. Perreault N. N. Niederberger T. D. Whyte L. G. Sun H. J. Leon R. (2008). Fluorescence microscopy as a tool for *in situ* life detection. Astrobiology 8, 859–874. 10.1089/ast.2007.0043 18752456

[B34] Orcutt B. N. Bach W. Becker K. Fisher A. T. Hentscher M. Toner B. M.. (2010). Colonization of subsurface microbial observatories deployed in young ocean crust. ISME J. 5, 692–703. 10.1038/ismej.2010.157 21107442 PMC3217339

[B35] Roussel E. G. Cambon Bonavita A. M. Querellou J. Cragg B. A. Webster G. Prieur D.. (2008). Extending the sub-sea-floor biosphere. Science 320, 1046–1046. 10.1126/science.1154545 18497290

[B36] Santelli C. M. Banerjee N. R. Bach W. Edwards K. J. (2010). Tapping the subsurface ocean crust biosphere: low biomass and drilling-related contamination calls for improved quality controls. Geomicrobiol. J. 27, 158–169. 10.1080/01490450903456780

[B37] Schijven J. F. Medema G. Vogelaar A. J. Hassanizadeh S. M. (2000). Removal of microorganisms by deep well injection. J. Contam. Hydrol. 44, 301–327. 10.1016/S0169-7722(00)00098-X

[B38] Schrenk M. O. Huber J. A. Edwards K. J. (2010). Microbial provinces in the subseafloor. Ann. Rev. Mar. Sci. 2, 279–304. 10.1146/annurev-marine-120308-081000 21141666

[B39] Stein C. A. Stein S. (1994). Constraints on hydrothermal heat flux through the oceanic lithosphere from global heat flow. J. Geophys. Res. 99, 3081–3081. 10.1029/93JB02222

[B40] Tong M. Camesano T. A. Johnson W. P. (2005). Spatial variation in deposition rate coefficients of an adhesion-deficient bacterial strain in Quartz Sand. Environ. Sci. Technol. 39, 3679–3687. 10.1021/es048850s 15952372

[B41] Tufenkji N. (2007). Modeling microbial transport in porous media: traditional approaches and recent developments. Adv. Water Resour. 30:1455–1469. 10.1016/j.advwatres.2006.05.014

[B42] Whitman W. B. Coleman D. C. Wiebe W. J. (1998). Prokaryotes: the unseen majority. Proc. Natl. Acad. Sci. U.S.A. 95, 6578–6583. 10.1073/pnas.95.12.6578 9618454 PMC33863

[B43] Yao K. M. Habibian M. T. O'Melia C. R. (1971). Water and waste water filtration. Concepts and applications. Environ. Sci. Technol. 5, 1105–1112. 10.1021/es60058a005

