# Supplementary material for: In situ Detection of Microbial Life in the Deep Biosphere in Igneous Ocean Crust
Source: Front Microbiol. 2015 Nov 12;6:1260. doi: 10.3389/fmicb.2015.01260 (PMC4641887; doi:10.3389/fmicb.2015.01260)
Supplement: Supplementary file 2 [file DataSheet1.DOCX]

**Table S1 DEBI-T Tool Specifications**

| Temperature Rating | Up to 50 ^0^C |
| --- | --- |
| Pressure Rating | 10,000 psi |
| Power Requirements | 24VDC, 2A |
| Length | 94” |
| Weight |  |
| Air | 53.5 Kg |
| Internal Components | 6 Kg |
| Submerged | 44.5 Kg |
| Detectors |  |
| Type | Photomultiplier Tubes (PMTs) |
| Number | 7 |
| Excitation Source | 224 nm HeAg Laser (Photon Systems, Inc.) |
| Laser Spot Size | 1mm from instrument – 2 mm ø |
| Video | High Definition pin-hole camera with microSD card capable of recording up to 8 hours of continuous video |
| Maximum Logging Speed | 275 m/hr (900 ft/hr) |
| Resoultion at Maximum Logging Speed | Data Point Every 15 mm |
| Sampling Rate | 4 Hz |
| Cablehead Connection | Schlumberger type |

**Supplementary Materials**:

**Figure S1.** The strategy for collecting fluorescence data from the borehole sidewall involved using an externally located quartz turning mirror to direct the laser excitation towards the wall. The excitation source exited DEBI-t through a downhole-facing, deep UV transmissive sapphire window. A long depth of focus allowed for the greatest flexibility for detection due to the dynamic nature of DEBI-t’s location with respect to the sidewall.

**Figure S2.** The microbiology combination tool string used during IODP Expedition 336 collected measured several physical parameters to correlate DEBI-t data to lithological and physical information about Hole 395A. A) From downhole to uphole: DEBI-t = Deep Exploration Biosphere Investigative tool; MFTM = Multifunction Telemetry Module; ELIC = EFTB-Lamont Interface Cartridge; MTT = Modular Temperature Tool; GPIT = General Purpose Inclinometry Tool; HNGS = Hostile environment Natural Gamma ray Sonde. B) Schematic of the deployment strategy for the microbiology combo tool in 395A. A complete run contained a downlog through the seafloor, an uplog to the base of the pipe, a downlog from pipe to the bottom of the hole, and a final uplog ending a few meters above seafloor.

**Figure S3.** The spectral characteristics of Mid-Atlantic Ocean Water are distinct from the signals collected within Hole 395A. Left) subset of data collected within 395A; Center) signal from 350 meters below seafloor; Right) spectra for Mid-Atlantic Ocean Water. Ocean water was collected during IODP 336 operations at North Pond.

**Figure. S4.** Bench set up for collecting library data used to classify signals from 395A. Data was collected at two different locations, right in front of the turning mirror and 10 inches from the turning mirror. This enabled a comparison of the collected spectra in front and behind the focal point.

**Movie M1.** This movie is a clip from DEBI-t’s first downhole pass within Hole 395A. The movie indicates that presence of a large number of particulates as well as white, floc-like material. This material makes the hole very cloudy, and reduces the likelihood that DEBI-t collected any significant signal from the borehole sidewall. Nevertheless, the detection of microbial-like signatures does suggest the presence of a significant biota within 395A.
