# Supplementary figures and images for: In situ Detection of Microbial Life in the Deep Biosphere in Igneous Ocean Crust
Source: Front Microbiol. 2015 Nov 12;6:1260. doi: 10.3389/fmicb.2015.01260 (PMC4641887; doi:10.3389/fmicb.2015.01260)

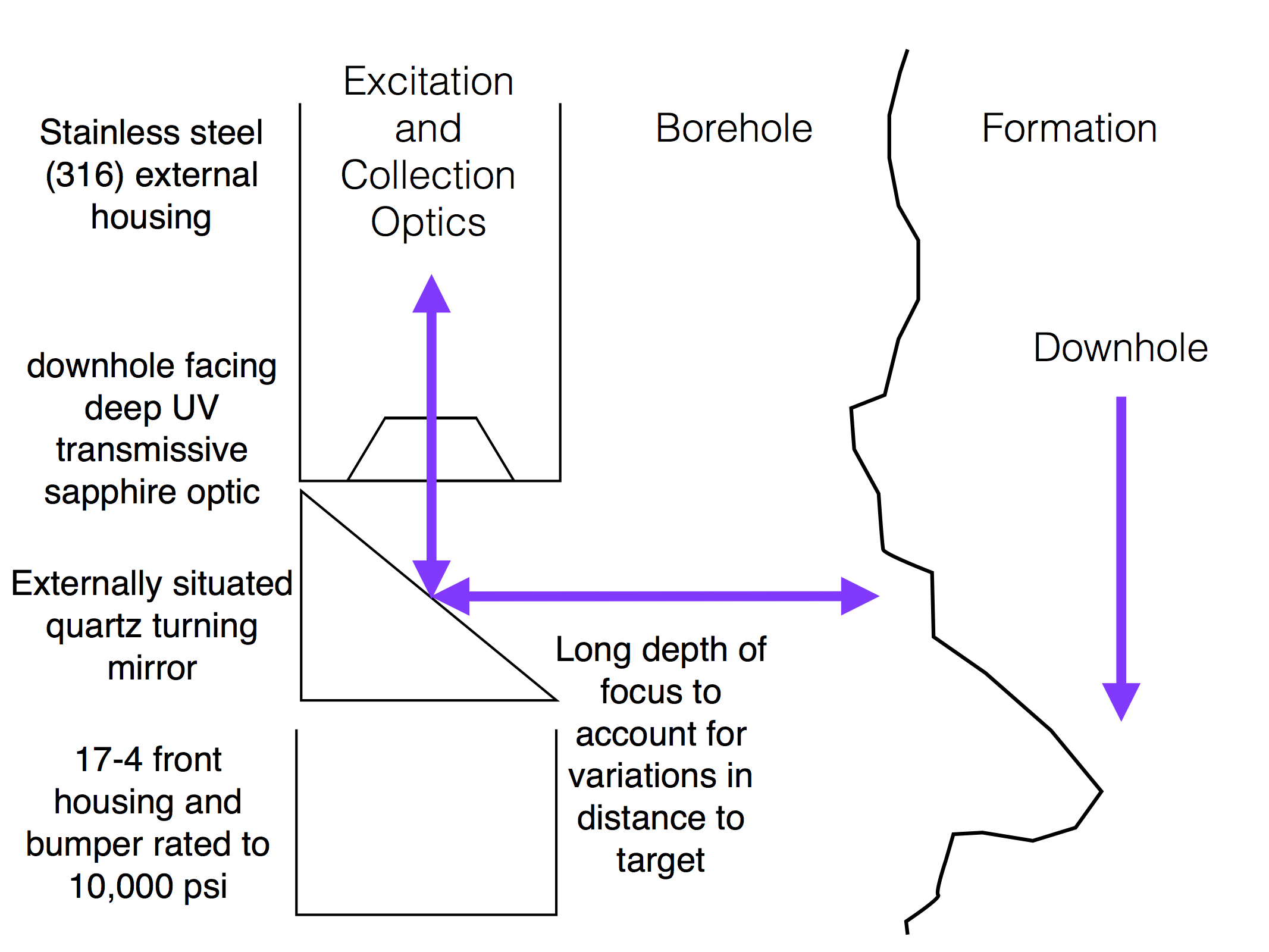

Supplement: Supplementary file 3 [file Image1.JPEG]

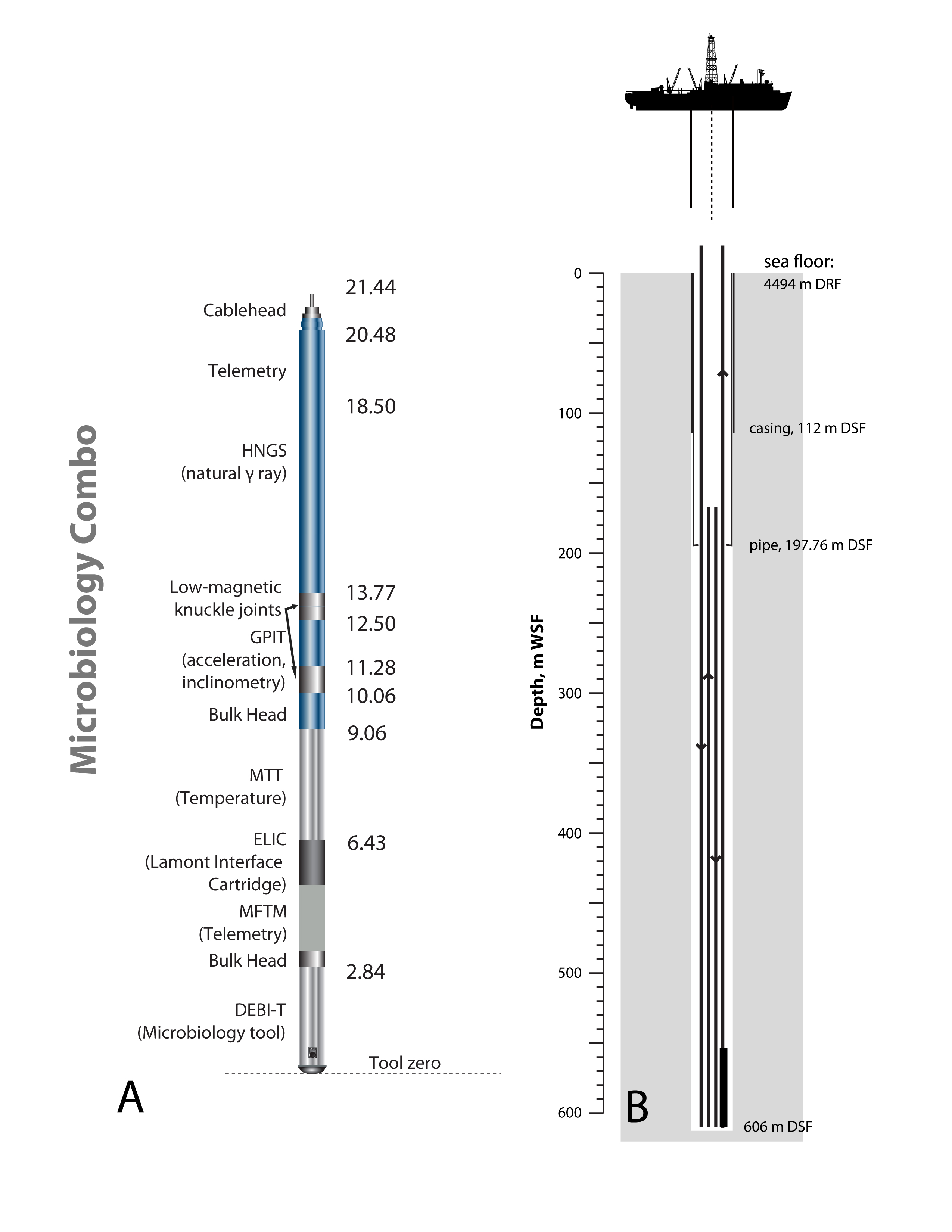

Supplement: Supplementary file 4 [file Image2.JPEG]

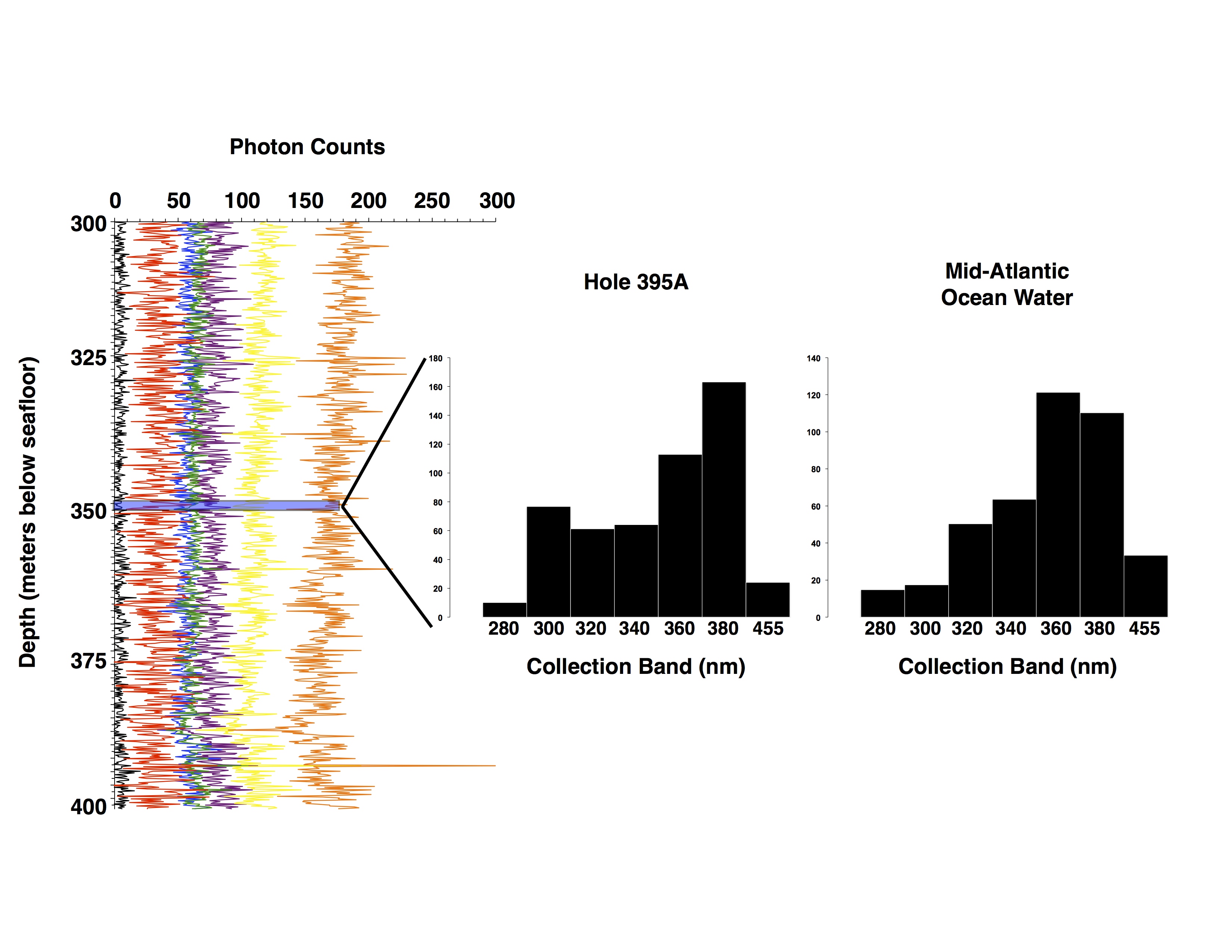

Supplement: Supplementary file 5 [file Image3.JPEG]

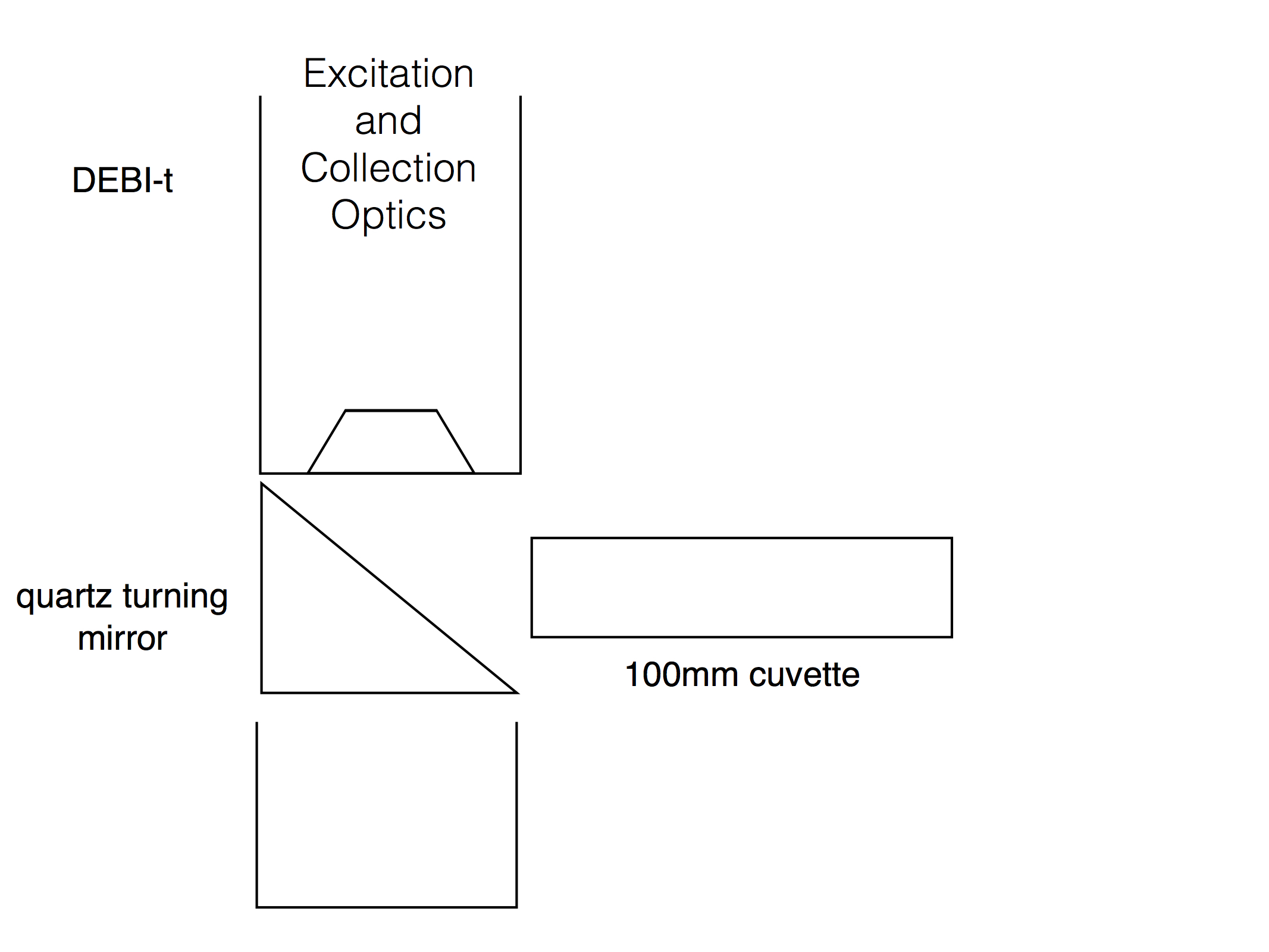

Supplement: Supplementary file 6 [file Image4.JPEG]
